# Primary and Malignant Cholangiocytes Undergo CD40 Mediated Fas Dependent Apoptosis, but Are Insensitive to Direct Activation with Exogenous Fas Ligand

**DOI:** 10.1371/journal.pone.0014037

**Published:** 2010-11-17

**Authors:** Elizabeth H. Humphreys, Kevin T. Williams, David H. Adams, Simon C. Afford

**Affiliations:** Centre for Liver Research, MRC Centre for Immune Regulation, The Institute of Biomedical Research, University of Birmingham, Birmingham, United Kingdom; Roswell Park Cancer Institute, United States of America

## Abstract

**Introduction:**

Cholangiocarcinoma is a rare malignancy of the biliary tract, the incidence of which is rising, but the pathogenesis of which remains uncertain. No common genetic defects have been described but it is accepted that chronic inflammation is an important contributing factor. We have shown that primary human cholangiocyte and hepatocyte survival is tightly regulated via co-operative interactions between two tumour necrosis family (TNF) receptor family members; CD40 and Fas (CD95). Functional deficiency of CD154, the ligand for CD40, leads to a failure of clearance of biliary tract infections and a predisposition to cholangiocarcinoma implying a direct link between TNF receptor-mediated apoptosis and the development of cholangiocarcinoma.

**Aims:**

To determine whether malignant cholangiocytes display defects in CD40 mediated apoptosis. By comparing CD40 and Fas-mediated apoptosis and intracellular signalling in primary human cholangiocytes and three cholangiocyte cell lines.

**Results:**

Primary cholangiocytes and cholangiocyte cell lines were relatively insensitive to direct Fas-mediated killing with exogenous FasL when compared with Jurkat cells, which readily underwent Fas-mediated apoptosis, but were extremely sensitive to CD154 stimulation. The sensitivity of cells to CD40 activation was similar in magnitude in both primary and malignant cells and was STAT-3 and AP-1 dependent in both.

**Conclusions:**

1) Both primary and malignant cholangiocytes are relatively resistant to Fas–mediated killing but show exquisite sensitivity to CD154, suggesting that the CD40 pathway is intact and fully functional in both primary and malignant cholangiocytes 2) The relative insensitivity of cholangiocytes to Fas activation demonstrates the importance of CD40 augmentation of Fas dependent death in these cells. Agonistic therapies which target CD40 and associated intracellular signalling pathways may be effective in promoting apoptosis of malignant cholangiocytes.

## Introduction

Fas (CD95) is a prototypic death inducing receptor of the TNF receptor superfamily which induces death by apoptosis when engaged by its ligand FasL (CD178) [1], [2]. Fas is widely expressed in the heart, liver, spleen and thymus [3] and regulated expression of CD178 is critical to prevent uncontrolled Fas activation in B lymphocytes [4]. The liver expresses low levels of Fas constitutively which increase rapidly during inflammation rendering hepatocytes potentially susceptible to Fas-mediated death. Biliary cells in primary biliary cirrhosis (PBC) were originally reported to undergo apoptosis in response to FasL cross linking which contributes to progressive bile duct loss [5]. In addition to Fas, liver epithelial cells express other death receptors of the TNF Receptor superfamily (TNFR) in response to inflammation or infection including TRAIL receptors, TNFR1/2, and CD40 rendering them susceptible to apoptosis via several receptors [6].

CD40, a 50 kDa cell surface type I glycoprotein first discovered on B cells, lacks a classical death domain [7]. The cognate ligand for CD40, CD154 (CD40 ligand, CD40L, gp39, TRAP) does not bind other TNFR although it can interact with the soluble complement inhibitor C4b binding protein which may function as a competitive inhibitor of CD40 activation [8]. CD154 is a type II transmembrane protein that retains activity as a soluble trimer. It is expressed on the surface of several cell types including activated T lymphocytes and macrophages [9], [10].

CD40 on B cells regulates cell survival, proliferation and isotype class switching [11]. Patients with X-linked hyper IgM syndrome have a mutation in the CD154 gene, which is associated with enhanced susceptibility to *Pneumocystis Carinii*, *Cryptosporidosis* and development of biliary tract, liver and pancreatic tumours [12]. The presence of CD40 on the majority of carcinomas [13], its absence or low constitutive expression on normal tissue, and inducibility of CD40 by pro-inflammatory cytokines such as TNFα and IFN-γ indicate a potential role for CD40 in the development of malignancy at sites where there is chronic inflammation [14].

That there are approximately 27 TNFR compared with 19 known TNF ligands implies shared receptors and integrated signaling pathways; for example all members of the TNF superfamily can activate NF-κB albeit to varying degrees [15]. We were the first group to demonstrate that CD40 activation induces Fas-dependent apoptosis of hepatocytes via induction of autocrine/paracrine FasL *in vitro*, and may amplify hepatocyte loss in liver injury [16]. Our subsequent work demonstrated that CD40 and Fas are expressed by cholangiocytes (biliary epithelial cells) in inflamed portal tracts and ligation of cholangiocyte CD40 by CD154 leads to autocrine or paracrine amplification of Fas mediated apoptosis mediated through sustained activation of AP-1 (JNK/ERK) and JAK2 dependent activation of STAT-3 [17], [18].

Cholangiocarcinoma is a rare but rapidly increasing malignancy of the biliary tract [19]. It is usually diagnosed late and average 5 year survival rates are only 5%–10%. Cholangiocarcinoma is 10 times more common in Japan and Southeast Asia due to a high incidence of *Clonorchis sinensis* and *Clonorchis viverrini* parasitic infections [20]. Other risk factors for cholangiocarcinoma include hepatolithiasis [21], abnormal biliary anatomy (15% increased risk) [22], chronic hepatitis B or C infection [23] and primary sclerosing cholangitis, a chronic inflammatory and scarring disease of the bile ducts [24]. The pathogenesis of cholangiocarcinoma development in biliary disease is unknown but chronic inflammation, bile salt toxicity and chronic bacterial contamination have all been implicated [25].

Constitutive activation of the CD40 pathway has been implicated in cell transformation and neoplastic growth [26]. Paradoxically some ovarian and cervical cancer cell lines express CD40 and can be induced to undergo apoptosis when stimulated with a range of TNF ligands [27]. This prompted us to investigate whether CD40 is expressed in cholangiocarcinoma and if so whether it is functional and therefore a potential therapeutic target.

We report that cholangiocarcinoma cells express functional CD40 and undergo apoptosis when stimulated with soluble trimeric CD154. This was associated with an increase in FasL mRNA and signaling via AP-1 (c-Fos/c-Jun) and STAT-3. We also show for the first time that both cholangiocarcinoma cell lines and primary cholangiocytes were comparatively insensitive to directly stimulation with exogenous FasL despite expressing abundant levels of cell surface Fas. These observations shed further light on TNFR mediated mechanisms which control epithelial cell fate and have important implications for therapeutic approaches to treatment of cholangiocarcinoma.

## Materials and Methods

### 2.1. Sources of liver tissue and isolation of cholangiocytes

Cholangiocytes were isolated from a variety of end stage liver disease tissue removed at transplantation and from non-diseased liver tissue from organs that were surplus to transplant requirements [28]. All tissue was obtained from fully informed patients with written consent. Ethical approval for the study was granted by the South Birmingham Research Ethics Committee (LREC ref. CA/5192). Liver tissue underwent mechanical and enzymatic digestion with Collagenase type 1A (Sigma Aldrich, Dorset, UK) and density gradient centrifugation on 33%/77% Percoll. Cholangiocytes were further purified by immunomagnetic isolation using antibodies against the cholangiocyte-specific receptor HEA 125 (Progen, Heidelberg, Germany). The cells were cultured in Dulbecco's Modified Eagle medium, Hams F12 plus 10% heat-inactivated human serum, glutamine (2 mM), hepatocyte growth factor (10 ng/ml, Peprotech, London, UK), epidermal growth factor (10 ng/ml, Peprotech), hydrocortisone (2 µg/ml), cholera toxin (10 ng/ml, Sigma Aldrich), tri-iodo-thyronine (2 nM, Sigma Aldrich) and insulin (0.124 U/ml) with penicillin (100 IU/ml) and streptomycin (100 µg/ml, Gibco, California, USA). Cells were cultured to confluence in tissue culture flasks coated with rat tail collagen.

Cells were harvested by gentle trypsinization (Invitrogen) and used between passage 2 and 5 to ensure phenotypic stability.

AKN-1 (a gift from Dr A Nussler, University of Ulm, Germany), CC-LP-1 and CC-SW-1 (a gift from Dr P Bosma, AMC Liver Centre, University of Amsterdam, Netherlands) were maintained in DMEM with 10% FCS, L-glutamine (2 mM), penicillin (100 U/ml) and streptomycin (100 µg/ml) on uncoated flasks. Jurkat cells were maintained in RPMI 1640 (Invitrogen) with 10% FCS, L-glutamine (2 mM), penicillin (100 U/ml) and streptomycin (100 µg/ml).

### 2.2 Expression of CD40, Fas and FasL by flow cytometry

Cell surface expression of CD40, Fas or FasL was assessed by flow cytometry using a Coulter Epics XL flow cytometer and the following antibodies: CD40-PE (10 µg/ml, Axxora, Bingham, UK), Fas-PE (10 µg/ml, Dako, Ely, UK) and FasL-PE (4 µg/ml, BD Pharmingen, Oxford, UK) with an isotype-matched IgG_1_-PE antibody (10 µg/ml, Dako). All data were analysed using Cytomation Summit v3.1 (Dako).

### 2.3 Assessment of FasL involvement in apoptosis by RT-PCR

Cholangiocyte FasL mRNA expression was measured by RT-PCR after incubation of cells with soluble recombinant human (srh) trimeric CD154 (1 µg/ml, Axxora, ALX-522-015), or soluble recombinant human FasL (50 ng/ml, Axxora, ALX-522-001) for 24 hours. The concentrations of CD154 and FasL were optimum as used in previously published studies [16], [17], [18]. The ligand concentrations were determined by titration against primary hepatocytes or cholangiocytes (for CD154) or against a Fas^high^ Jurkat cell line. No further increases in apoptosis was observed with higher concentrations of either ligand on the primary cells (data not shown) or on the Jurkat cell line where 50 ng/ml result in apoptosis of >90% of the total population. Total RNA was extracted with the Qiagen RNEasy kit (Qiagen, Crawley, UK) according to the manufacturer's protocol. FasL and β-actin mRNA were detected using RT-PCR. Primer pairs for FasL and β-actin were synthesized by Alta Bioscience Ltd. (Birmingham, UK) using the following sequences: FasL forward 5′-ACTGGCAGCATCTTCACTTC-3′ and reverse 5′-TTCAAAATCTTGACCAAATGC-3′, β-actin forward 5′-CATCACCATTGGCAATGAGC-3′ and reverse 5′-ATCCACACGGAGTACTTG-3′. Reverse transcription (RT) was carried out using a standard protocol incubating the RT mix at room temperature for 10 min, followed by 1 h at 42°C and 5 min at 95°C. Samples were held at 4°C before PCR amplification according to the following cycling program: 2 min denaturation at 96°C, followed by 35 cycles of 30 seconds at 96°C, 1 minute at 55°C, and 1 minute at 72°C with a final extension for 10 minutes at 72°C. Samples were held at 4°C overnight. After PCR amplification, samples were electrophoresed in 2% agarose gel and stained with ethidium bromide.

### 2.4 Transcription factor activation in cells following stimulation with CD154

Cholangiocyte monolayers were treated with srh CD154 (1 µg/ml), srh FasL (50 ng/ml) or srh TNFα (10 ng/ml Peprotech) for 24 hours. Monolayers were then harvested by scraping into ice cold PBS. Cytoplasmic and nuclear protein extracts were prepared using a standard protocol [29] and protein content of each sample determined using the Micro Lowry Total Protein Kit (Sigma, according to the manufacturer's instructions). Samples (40 µg protein) were resolved on a 10% Bis-Acrylamide gel by SDS-PAGE, followed by transfer to nitrocellulose membrane (Amersham Pharmacia Biotech, Buckinghamshire, UK). Membranes were blocked overnight at 4°C in phosphate-buffered saline (PBS) containing 5% w/v non-fat dried milk and then washed (3×10 minutes) with 0.1% Tween-20/PBS, before incubating with primary antibodies. All incubations were for 1 hour at room temperature in PBS Tween containing 5% w/v non-fat dried milk. The following antibodies were used; a) phospho-STAT3 mouse monoclonal antibody at 0.2 µg/ml (Santa Cruz Biotechnology Inc. Cat. No. sc-8059, Santa Cruz, CA, USA). b) c-Fos rabbit polyclonal primary antibody at 0.4 µg/ml (Santa Cruz Biotechnology Inc. Cat. No. sc-52). c) c-Jun rabbit polyclonal primary antibody at 0.4 µg/ml (Santa Cruz Biotechnology Inc. Cat. No. sc-44) and d) β-actin mouse monoclonal antibody at 0.4 µg/ml (Sigma, Cat. No. A5441). Antibodies were detected using either horse radish peroxidase (HRP) conjugated rabbit anti mouse IgG (1.3 µg/ml) for 1 hour at room temperature or HRP conjugated goat anti rabbit IgG (0.25 µg/ml) for 1 hour at room temperature (Dako). Protein bands were visualised using enhanced chemiluminescence (Pierce Perbio, Cramlington, UK) and exposure to Hyperfilm-ECL (Amersham Pharmacia Biotech) and quantified by densitometry scanning (BioRad 2000 Gel Doc system, BioRad, Hemel Hempsted, UK). Equality of protein loading and transfer onto membranes was confirmed by probing for β-actin and staining with Ponceau S solution (Sigma).

### 2.5 Assessment of apoptosis by in situ DNA end labelling (ISEL) staining

Apoptosis was quantified by ISEL staining and assessment of morphology on cytospin preparations of cholangiocytes as previously described [30], [31].

Cholangiocytes were incubated for 24 hours in a) medium alone b) 1 µg/ml srh CD154, a concentration previously shown to provide optimal induction of apoptosis in these cells [15] or srh FasL 50 ng/ml (optimal dose for induction of apoptosis in the Jurkat cell line).

### 2.6 Statistical analysis

Data are expressed as the mean ± SD from at least three experiments unless otherwise stated. A Student's unpaired t-test was used to compare individual data with control values. A probability of *P*<0.05 was taken as denoting a significant difference from control data. All primary cell culture experiments were carried out a minimum of 3 times.

## Results

### Protein expression of CD40, Fas and FasL on isolated cholangiocytes

**Figure 1:**
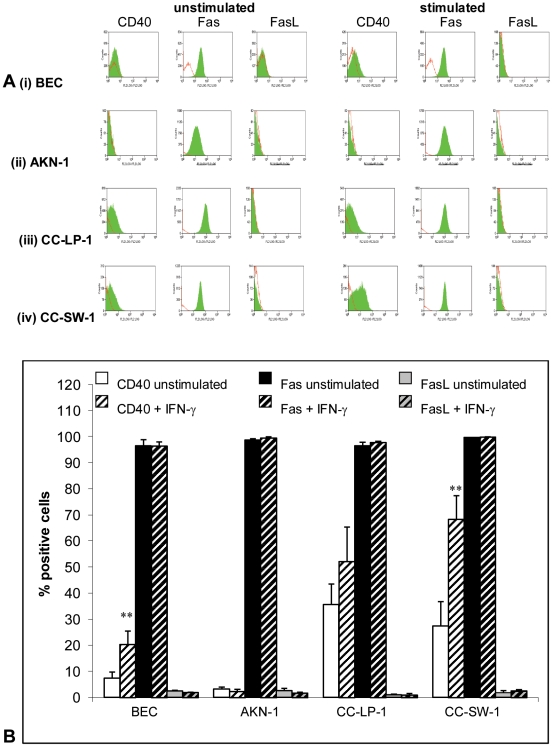
Expression of CD40, Fas and FasL protein on the cell surface assessed by flow cytometry. Cells were grown in media alone or stimulated with IFN-γ at 50 ng/ml for 48 hours. (a) flow cytometry histograms of matched isotype control (red outline) and CD40, Fas and FasL expression (green solid colour) (b) shows the percentage positive cells for CD40, Fas and FasL protein. n = 3+/− standard deviation. *p<0.05, **p<0.01 as compared to untreated controls.

In the absence of stimulation cultured primary human cholangiocytes and the cell lines expressed CD40, high levels of Fas and very low levels of FasL. Fas and FasL levels were unchanged by the addition of IFN-γ to the culture media for 48 hours whereas CD40 expression increased significantly in response to IFN-γ in all cell types except AKN-1 cells (figure 1).

### Induction of FasL mRNA following CD40 activation on cholangiocarcinoma cell lines and primary cholangiocytes

**Figure 2:**
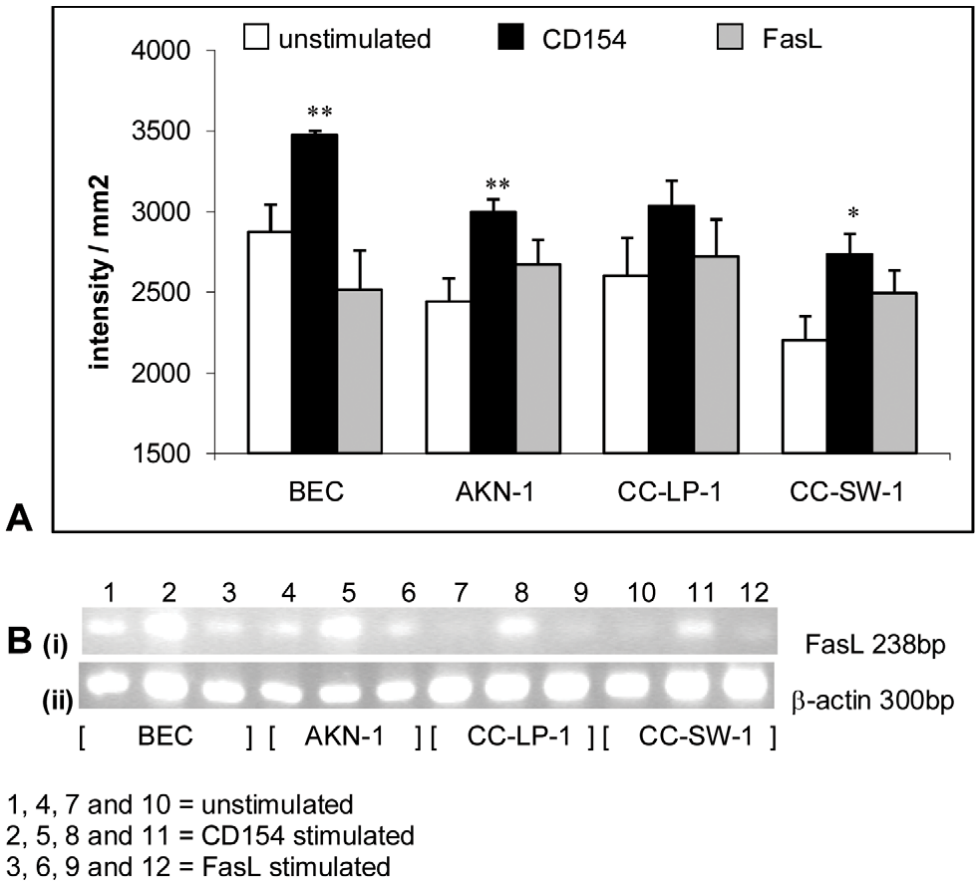
Fas ligand induction to promote apoptosis assessed by RT-PCR at 4 hours. Cells were grown in culture media alone, stimulated with srh CD154 at 1 µg/ml or srh FasL at 50 ng/ml for 4 hours. (a) densitometry scanning data for FasL PCR gels at 4 hours. n = 4+/− standard error of the mean. (b i) a representative FasL PCR gel and (b ii) the corresponding β-actin gel. *p<0.05, **p<0.01 as compared to untreated controls.

Activation of cholangiocarcinoma cell lines or primary cholangiocytes with soluble trimeric CD154 resulted in a significant increase in FasL mRNA after 4 hours stimulation compared with basal levels in unstimulated cells. Although the results for the CC-LP-1 cell line failed to reach statistical significance (when compared to β-actin controls) the trend was the same as the other two cell lines and the primary cells (figure 2).

### Transcription factor activation in cholangiocarcinoma cells compared with primary cholangiocytes

**Figure 3:**
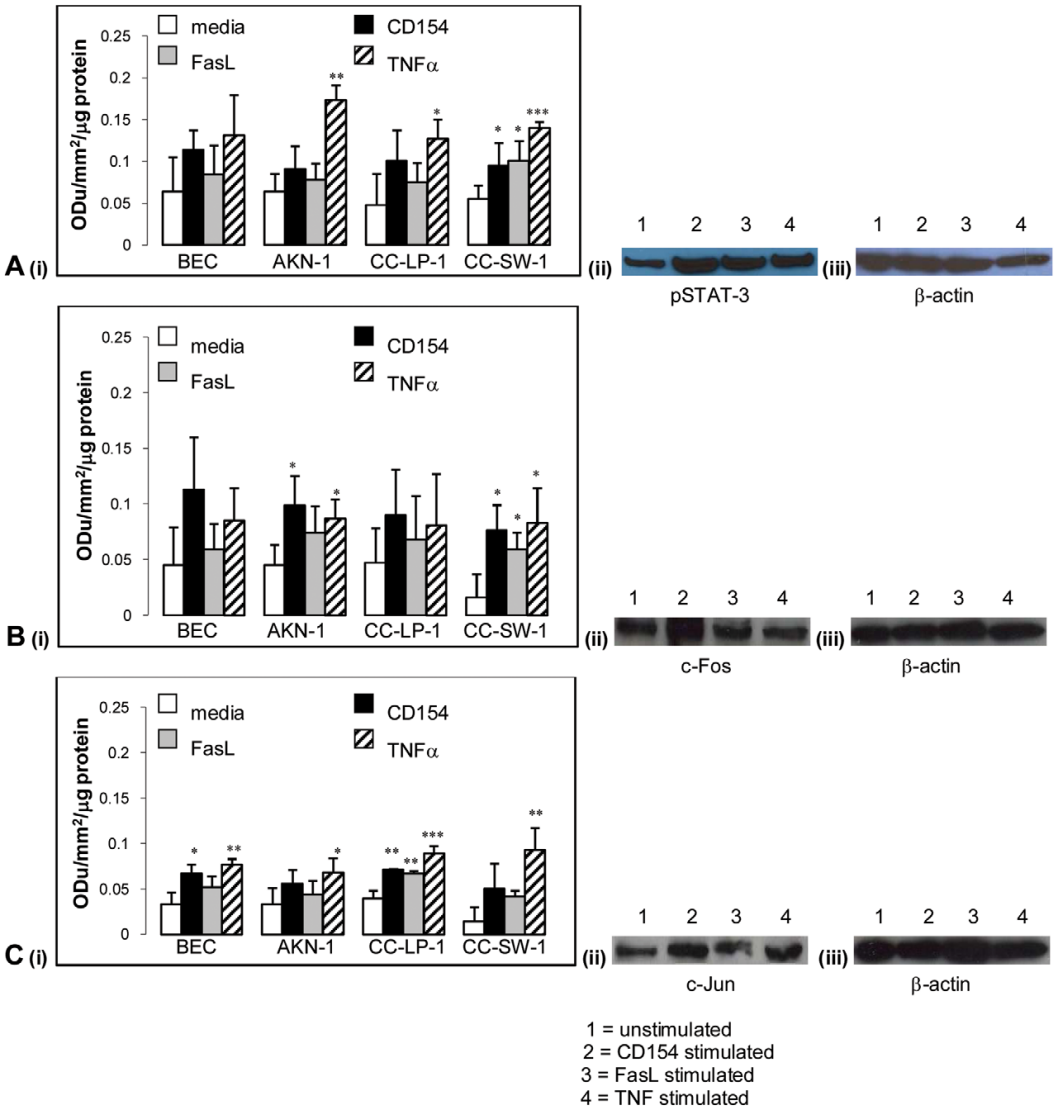
Transcription factor activation in cells undergoing apoptosis after stimulation with soluble TNF ligands. Cells were grown in media alone, with srh CD154 at 1 µg/ml, srh FasL at 50 ng/ml or srh TNFα at 10 ng/ml for 24 hours. Graphs show densitometry scanning data, n = 3+/− standard deviation and representative autoradiographs of primary cholangiocyte samples showing (a) pSTAT-3 (60 kDa), (b) c-Fos (60 kDa) and (c) c-Jun (35 kDa) all with their corresponding β-actin blots (45 kDa). *p<0.05, **p<0.01, ***p<0.001 as compared to untreated controls.

We previously reported [17] that activation of cholangiocytes by CD40 ligation results in sustained upregulation of c-Fos/c-Jun (AP-1 subunits) and phosphorylation of STAT3 and apoptotic cell death [18]. Here although there was some variation in the magnitude of response between cells types, the trend/activation profile was similar to the primary cells in all three cholangiocarcinoma cell lines (figure 3). pSTAT-3, c-Fos and c-Jun increased in all cells following stimulation with CD154 compared to unstimulated cells or those stimulated with FasL or TNFα.

### Induction of apoptosis after stimulation with soluble CD154 or FasL

**Figure 4:**
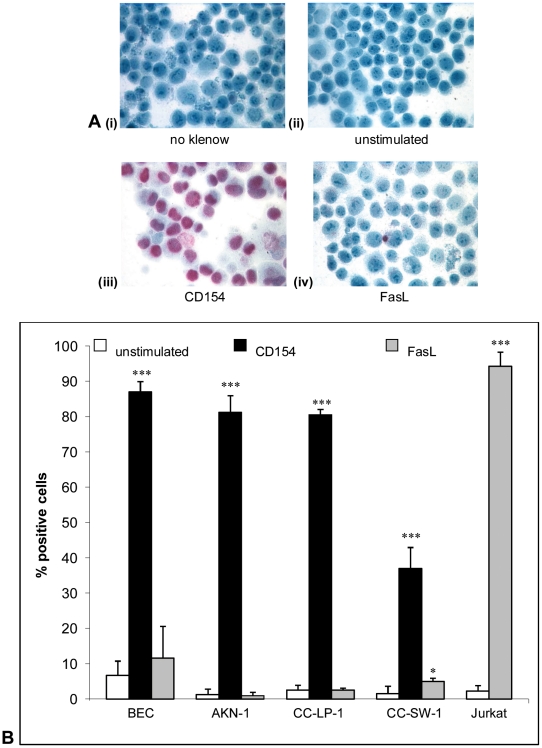
Apoptosis induction in cholangiocytes after stimulation with soluble TNF ligands. All cells were cytocentrifuged and stained by the standard ISEL technique to detect apoptosis after culture in media alone, media with srh CD154 at 1 µg/ml or media with srh FasL at 50 ng/ml for 24 hours. All control slides without DNA polymerase klenow fragment were negative (a i). (a ii) primary cholangiocytes in media alone, (a iii) shows primary cholangiocytes stimulated with soluble CD154 and (a iv) primary cholangiocytes challenged with soluble FasL. (b) shows the percentage positive ISEL stained cells in all cell types when grown in media alone, with CD154 or with FasL. n = 3+/− standard deviation. *p<0.05, **p0.01, ***p<0.001 as compared to untreated controls.

Activation of cell-surface CD40 with srh CD154 induced similar high levels of apoptosis in primary and malignant cells after 24 hours; whereas only the CC-SW-1 cholangiocarcinoma cell line underwent apoptosis when stimulated with FasL for 24 hours despite all cell lines and primary cells expressing high levels of cell surface Fas. The Jurkat cell line was used as a positive control to show that the srh FasL was functional and could induce apoptosis in an appropriately sensitive cell line cultured in similar conditions (figure 4).

## Discussion

We have demonstrated that cholangiocarcinoma cells express CD40 and like primary cholangiocytes are exquisitely sensitive to CD154 mediated apoptosis. After stimulation with CD154 there was a sustained upregulation of FasL and induction of apoptosis was accompanied by activation of the AP-1 (c-Fos/c-Jun) and pSTAT-3 signalling pathways as previously reported in primary cholangiocytes [16], [17], [18]. Thus the functional elements of the CD40 death-inducing mechanism are intact in cholangiocarcinoma.

The expression of CD40 on cholangiocarcinoma and the increased levels of expression detected after IFN-γ stimulation are consistent with previous studies reporting CD40 expression on other carcinomas e.g. nasopharyngeal carcinoma [32], [33]. The increased levels of CD40 seen in diseased tissue and cholangiocarcinoma could be explained by high local levels of proinflammatory cytokines such as TNFα and IFN-γ at sites of persistent chronic inflammation [14].

Fas is expressed by non-malignant bile ducts and expression increases in chronic inflammatory biliary disease and on bile ducts that have become dysplastic [34]. Fas is also expressed in well-differentiated cholangiocarcinoma but expression is diminished or absent from poorly differentiated tumours [34]. Cholangiocarcinoma cell lines display variable levels of Fas, for example the extrahepatic cholangiocarcinoma cell line Sk-ChA-1 includes a mixed population of high and low Fas-expressing cells as determined by mRNA and protein analysis [35], [36]. Thus although cholangiocytes constitutively express Fas and this is preserved during neoplastic transformation it can be downregulated as the tumour becomes more genetically unstable [34].

FasL mRNA expression has also been reported to be increased in malignant cholangiocytes compared with the low levels detected in non-malignant cells [37]. All three cell lines we studies showed low expression of FasL but this was readily increased by CD154 stimulation. Inhibition of gene transcription, alteration of posttranscriptional modification and protein processing may account for the low constitutive FasL expression observed.

After 4 hours incubation in media alone or with srh FasL, low levels of FasL mRNA were detected. When cells were incubated with soluble recombinant CD154, expression of FasL mRNA was markedly upregulated. These data are consistent with previous studies in which CD154 stimulation of primary non-malignant cholangiocytes increased FasL mRNA threefold compared to media alone. Previous studies with hepatocytes also showed that apoptosis after stimulation with CD154 could be significantly reduced with a blocking antibody to FasL. The sustained upregulation of FasL may explain why the pro-apoptotic effects of CD154 are so potent providing an essential amplification signal to maintain FasL production and thus promote autocrine/paracrine Fas activation [17].

Specific transcription factors have been implicated in regulating cholangiocyte death and survival [17], [18] and here we found these were conserved in the cholangiocarcinoma cells. The signalling pathways examined previously were characterized extensively using specific pharmacological inhibitors and shown to be critical for induction of cholangiocyte apoptosis after CD154 ligation. STAT-3 was examined because it can regulate cell survival and drive apoptosis in mammary epithelial cells [38]. Previous studies in the liver suggested that STAT-3 is a global cell survival factor involved in tissue regeneration but these experiments used whole liver extracts and therefore do not show what happens to individual cell types [39]. It is the phosphorylated form of the molecule that conveys signals and at 24 hours after treatment with CD154 levels of phospho STAT-3 were increased compared with FasL stimulated or unstimulated cells suggesting that in these circumstances and cells STAT-3 is pro-apoptotic [18]. CD40 ligation can also cause the activation of the transcription factor complex AP-1, which consists of two heterodimer subunits c-Fos and c-Jun [40] and these have been found to be pro-apoptotic [41], [42], [43] because they form dimers capable of binding to promoter regions of genes involved in apoptosis such as caspase 3. Consistent with this we found increased levels of c-Fos and c-Jun in cells undergoing apoptosis at 24 hours in response to CD154 but not FasL. The activation demonstrated here of STAT-3 and AP-1 (c-Jun and c-Fos) following CD154 treatment suggests that these are common pathways downstream for CD40 in both the primary and malignant cells and are associated with apoptotic cell death. *In vivo* the scenario is likely to be more complex. It may be for example that cholangiocarcinoma cells do become increasingly insensitive to Fas ligation and protected from CD40 induced apoptosis as a consequence of the high levels of the CD154 antagonist C4BP found in close proximity to the tumours [8]. At present this remains an area under active investigation in our laboratory.

Primary cholangiocytes and cholangiocarcinoma cells intriguing seemed to express low levels of CD40 but high levels of Fas. However in contrast to CD154, very little apoptosis was observed when stimulated directly with FasL. This finding was not expected and suggests that Fas activation does not deliver a potent pro-apoptotic signal in cholangiocytes or their malignant counterparts. Fas resistance is well-reported in several cancers [44], [45] and may provide an important survival advantage to the tumour. However here we have demonstrated for the first time that normal cholangiocytes are similarly insensitive to FasL. The reasons for this are presently unknown but the fact that we can induce Fas-dependent apoptosis via CD40 ligation in these cells suggests that CD40 not only induces FasL expression to provide autocrine Fas activation but that it also directly induces signals that activate the otherwise suppressed Fas death pathway. The finding that the lymphocyte-derived Jurkat cell line readily underwent apoptosis when stimulated with srh FasL indicates that the differences were due to cell type and not the biological activity of the ligand.

When any of the cell types were challenged with soluble CD154, the majority underwent apoptosis, with the cell lines being equally as sensitive as the primary cells. The response of primary cholangiocytes is consistent with what we have shown previously [17] but differ from a study which reported that both non-malignant and malignant urothelial cells failed to undergo apoptosis when stimulated with soluble CD154, whereas cell-bound CD154 induced apoptosis of the malignant cell lines. This lead the authors to conclude that mode of ligand presentation was important for the induction of apoptosis [46]. That we saw responses with srh CD154 might relate to the nature of the ligand we used but suggests also that ligand presentation may not be critical for this pathway.

At least one previous study reported that a CD40 positive tumour cell line resistant to Fas cytotoxicity was susceptible to TNFR or CD40-mediated death [47]. It has been known for a number of years that members of the TNF superfamily can interact with each other, and this includes CD40 and Fas. While some studies showed that stimulating the CD40 receptor inhibits Fas-induced apoptosis in normal and neoplastic cells [48], [49], many more have shown that CD40 and Fas can synergize to enhance the apoptosis signal to increase cell death and that receptor levels on the cell surface may determine the outcome. Other studies have examined the CD40/Fas costimulatory effects on apoptosis in malignant cells and found that this also occurs in a range of tumour cell lines including the human myeloma cell line XG2 [50], B cell lymphoma cells [51], breast carcinoma cell lines [52] and chronic lymphocytic leukemia cells [53]. Chu and colleagues reported that CD40 ligation on CLL B cells induced a programme of events in which shifts in the balance of anti-apoptotic and pro-apoptotic FLIP and FADD were associated with initial resistance to Fas-mediated killing followed by subsequent sensitization. A similar mechanism could be operating in these cholangiocarcinoma cells that are resistant to direct Fas ligation but die in a Fas-dependent process after CD40 ligation. Here we have shown that similar mechanisms apply to cholangiocarcinoma cells.

One of the most interesting findings in this study was that despite the apparent low levels of CD40 on the cell surface, all of the cholangiocarcinoma cell lines nevertheless under went high levels of apoptosis. This apparent lack of correlation between receptor levels and biological effect may be explained by differences in receptor density and/or occupancy. The importance of receptor occupancy and CD154 concentration in determining functional outcome has been reported previously in Burkitt lymphoma cells where high concentrations of CD154 suppressed proliferation while low concentrations of CD154 sustained growth [54]. Another and possibly more likely explanation was that only a small subpopulation of cells are required to express CD40 which once stimulated express and present sufficient FasL to drive apoptosis in Fas expressing neighboring cells in a juxtacrine or paracrine manner. This clearly warrants further studies which are beyond the scope of the present investigation but if correct it suggests that under these circumstances it is the juxtacrine/paracrine mechanism of FasL presentation which predominates as a mechanism for regulation of apoptosis in cholangiocytes. This remains speculative but worthy of future investigation.

Because CD40 is upregulated in many cancers it is a potential target for anti-cancer therapy. Recombinant CD154 has been used in a phase I clinical trial in patients with either advanced solid tumours or high grade non-Hodgkin's lymphoma with some evidence of efficacy and little evidence of serious toxicity [55]. A humanized anti-CD154 antibody (hu5C8) was developed with the objective of suppressing CD40-mediated immune activation in autoimmune disease and transplantation but clinical trails were complicated by increased thromboembolic events and had to be halted [56]. Anti-human CD154 antibody (AB1793) has subsequently been shown to prevent kidney allograft rejection in monkeys presumably by suppression of immune cell activation [57] but also possibly by inhibiting renal epithelial cell loss resulting from similar mechanisms to those proposed in the liver. The authors concluded that the thromboembolic events seen previously were antibody class-specific thus reviving enthusiasm for its clinical use.

Tumours which express CD40 can stimulate the generation of anti-tumour effector cells [58] and CD40 activation *in vivo* enhances the survival of activated T cells [59] and promotes DC accumulation and survival [60]. CD154 has been transduced into murine tumours in a mouse model of prostate cancer to reduce cell viability and increase apoptosis. Vaccination of CD154-expressing cells induced anti-tumour immunity and peritumoural injections induced tumour growth suppression [61]. There is growing evidence that macrophages are capable of killing tumour cells [62] and our previous studies reporting that CD154-bearing macrophages are present in the human liver and can induce apoptosis in CD40-expressing cholangiocytes [63] suggests another mechanism through which CD40 could mediated anti-tumour responses in cholangiocarcinoma.

In this study we have shown that cholangiocarcinoma cells lines are resistant to Fas–mediated killing but show sensitivity to CD40 mediated apoptosis suggesting that CD154 therapy might be effective in the treatment and management of cholangiocarcinoma. CD40/CD154 therapy in cholangiocarcinoma could work in two ways; by stimulating anti-tumour responses in leukocytes, and by the indirect promotion of cell death via CD40-dependent induction of FasL. However caution is necessary because there is the potential for inducing death of non-malignant cholangiocytes if the treatment is not targeted to the tumour.
